# Supplementation with long chain n-3 fatty acids during pregnancy, lactation, or infancy in relation to risk of asthma and atopic disease during childhood: a systematic review and meta-analysis of randomized controlled clinical trials

**DOI:** 10.29219/fnr.v66.8842

**Published:** 2022-10-11

**Authors:** Linnea Bärebring, Bright I. Nwaru, Christel Lamberg-Allardt, Birna Thorisdottir, Alfons Ramel, Fredrik Söderlund, Erik Kristoffer Arnesen, Jutta Dierkes, Agneta Åkesson

**Affiliations:** 1 Department of Internal Medicine and Clinical Nutrition, Institute of Medicine, Sahlgrenska Academy, University of Gothenburg, Gothenburg, Sweden; 2 Krefting Research Centre, Institute of Medicine, University of Gothenburg, Gothenburg, Sweden; 3 Department of Food and Nutrition, University of Helsinki, Helsinki, Finland; 4 Faculty of Sociology, Anthropology and Folkloristics, Health Science Institute, University of Iceland, Reykjavík, Iceland; 5 Faculty of Food Science and Nutrition, University of Iceland, Reykjavík, Iceland; 6 Unit of Cardiovascular and Nutritional Epidemiology, Institute of Environmental Medicine, the Karolinska Institute, Solna, Sweden; 7 Department of Nutrition, Institute of Basic Medical Sciences, University of Oslo, Oslo, Norway; 8 Centre for Nutrition, Department of Clinical Medicine, University of Bergen, Bergen, Norway; 9 Department of Laboratory Medicine and Pathology, Haukeland University Hospital, Bergen, Norway

**Keywords:** asthma, atopy, nutrition, fatty acids, omega 3

## Abstract

**Objective:**

To assess whether supplementation with long chain n-3 fatty acids during pregnancy, lactation, or infancy reduces the risk of developing asthma or atopic disease during childhood.

**Methods:**

Searches were performed in MEDLINE, Embase, Cochrane Central Register of Controlled Trials, and Scopus up to 2021-09-20, for randomized controlled trials (RCTs) that investigated the effect of supplemental long chain n-3 fatty acids during pregnancy, lactation, or infancy for the prevention of childhood asthma or allergy. Article selection, data extraction, and risk of bias assessment (Cochrane’s Risk of Bias 2.0) were independently conducted by two assessors. The evidence was synthesized qualitatively according to the criteria of the World Cancer Research Fund and meta-analyzed.

**Results:**

A total of nine RCTs met inclusion criteria; six were conducted during pregnancy, two during infancy, and one during both pregnancy and infancy. Meta-analysis showed that long chain n-3 fatty acid supplementation during pregnancy significantly reduced the risk of asthma/wheeze in the child (RR 0.62 [95% confidence interval 0.34–0.91], *P* = 0.005, *I*^2^ = 67.4%), but not other outcomes. Supplementation during lactation of infancy showed no effects on any outcome. The strength of evidence that long chain n-3 fatty acid supplementation during pregnancy reduces risk of asthma/wheeze in the offspring was considered *limited – suggestive*. No conclusion could be made for the effects of long chain n-3 fatty acid supplementation during pregnancy for other atopic diseases, or for supplementation during lactation or infancy for any outcome.

**Conclusion:**

The intake of long chain n-3 fatty acid supplements during pregnancy may reduce the risk of asthma and/or wheeze in the offspring, but the strength of evidence is low. There is inconclusive evidence for the effects of long chain n-3 fatty acid supplements during pregnancy for other outcomes, as well as for supplementation during lactation or infancy.

## Popular scientific summary

- This systematic review assessed whether the intake of omega three supplements during pregnancy, lactation, or infancy reduces atopic disease such as allergies or asthma in childhood.
- The results show that omega three supplements during pregnancy may reduce the risk of asthma or asthma symptoms during childhood, but the evidence is limited.
- No conclusions could be drawn for effects on other atopic diseases or for effects of supplementation during lactation of infancy.
- More high-quality research is needed to clarify if and how omega three supplements during pregnancy or infancy reduce risk of asthma or allergies during childhood.

The prevalence of atopic disease, including both asthma and allergic asthma, has increased in recent decades in the Western world, and asthma prevalence is approximately 10% in adult Nordic populations (1, 2). Nordic countries also have some of the world’s highest disability-adjusted life-years lost from atopic dermatitis (3). The increase in prevalence of asthma and atopic diseases has been ascribed to changes in environmental exposures, including dietary intake (4, 5).

Early life nutrition, i.e. nutrition during pregnancy and infancy, is linked to chronic disease risk later in life (6) and may also impact the risk of atopic disease and asthma in children (7). Omega three (n-3) polyunsaturated fatty acids (PUFAs), especially the long-chain n-3 polyunsaturated fatty acids (LCn3PUFAs), eicosapentaenoic acid (EPA), and docosahexaenoic acid (DHA), are nutritional factors of specific interest due to their immunomodulatory properties, caused by altered synthesis of bioactive lipid mediators and cell membrane composition (8, 9).

Observational studies show disparate findings regarding association between fish consumption in pregnancy or childhood and risk of asthma or other atopic diseases during childhood (10–14). Both observational and interventional studies show unclear evidence for LCn3PUFA supplementation during pregnancy or infancy for the prevention of childhood atopic disease (15, 16). Use of n3-supplements differs between the Nordic countries and is reported by a majority of pregnant women in Norway (17) and Iceland (18) and less frequently in other countries (19–21). Based on the uncertainties regarding the available evidence and new data published, the Nordic Nutrition Recommendations (NNR) Committee recommended that the topic of LCn3PUFA supplementation during pregnancy, lactation, or infancy for the prevention of atopic disease in the offspring be shortlisted for a systematic review in preparation for the 2022 edition of NNR (22).

The aim of this systematic review was to assess whether supplementation with LCn3PUFA during pregnancy, lactation, or infancy reduces the risk of asthma or atopic disease during childhood.

## Methods

This systematic review was conducted according to the guidelines for systematic reviews, developed for the 2022 revision of the NNR (23, 24) and Preferred reporting for systematic reviews (25). The NNR 2022 is funded by the Nordic Council of Ministers and governmental food and health authorities of Norway, Finland, Sweden, Denmark, and Iceland (26). A study protocol was published prior to article selection in the database PROSPERO (https://www.crd.york.ac.uk, CRD42021275309).

### Eligibility criteria

The research question was specified by the NNR 2022 Committee and the NNR Systematic Review Center (i.e. the authors) by defining the population, intervention/exposure, control, timing, study design, and setting (PI/ECOTSS).

As outlined in the PI/ECOTSS in Table 1, the intervention included supplemental intake of LCn3PUFA during pregnancy, lactation, or infancy, with placebo or other oils as comparator. Outcomes should have been assessed at age 0–18 years and were the following: asthma and/or wheeze, allergy (either allergic rhinitis, allergic sensitization, or specific allergies), and atopic dermatitis or eczema. Only randomized control trials (RCTs), with a ≥4 weeks intervention duration, were eligible for inclusion.

**Table 1:** Population, intervention/exposure, comparator, outcomes, timing, setting, and study designs (PI/ECOTSS) criteria for the papers to be included in the systematic review

Population	Intervention or exposure	Comparators	Outcomes	Timing	Setting	Study design
Pregnant and lactating women and their offspring	Supplemental intake of long chain n-3 fatty acids (fish oil, tran and pure marine n-3) Intervention during: pregnancy, lactation, or infancy (0–12 months)	Placebo or other oils	Asthma, wheeze, and allergies at 0–18 years of age To be included: - Asthma and/or wheeze - Allergy (allergic rhinitis, allergic sensitization, or allergies) - Atopic dermatitis or eczema	Minimum 4 week-intervention	Relevant for children and adolescents in the Nordic and Baltic countries	RCTs

n-3: omega 3; RCTs: randomized controlled trials.

### Search strategy

The literature searches were performed by research librarians from Karolinska Institute on 2021-09-20 in MEDLINE, Embase, Cochrane Central Register of Controlled Trials, and Scopus. The search strategy (Supplement 1) was developed in collaboration with the authors and peer reviewed by university librarians from the University of Oslo. Reference lists of relevant retrieved articles were also screened to identify additional articles. The searches utilized no restrictions on publication dates or language. Grey literature and unpublished studies were not searched.

### Article selection and data collection

Screening and selection of studies for inclusion/exclusion was performed by two authors (LB and CLA), working independently. The screening of titles and abstracts was performed in Rayyan (27). A pilot test was conducted using 10% of the titles and abstracts in order to harmonize the process. If at least one of the assessors voted for inclusion, a paper was selected for full text screening. Discrepancies were resolved by discussion with a third author (AÅ). Data from full-text papers included in the systematic review were extracted in standardized extraction forms by pairs of two authors working independently (LB, CLA, and FS).

### Risk of bias assessment

Risk of bias of each included study was assessed by two authors (BN and JD) working independently. The assessment tool used was Cochrane’s Risk of bias 2 (28). The risk of bias in each individual study was classified as low, high, or ‘some concerns’ for risk of bias, per outcome and population.

### Synthesis and strength of evidence

The evidence was synthesized qualitatively, based on study characteristics, context, strengths and limitations, heterogeneity, and relevance. In accordance with the guidelines for systematic reviews, meta-analyses were considered if deemed appropriate to combine the different studies, but only when more than three independent RCTs existed. We performed meta-analysis using a random-effects model to pool effect estimates from studies judged to be sufficiently homogeneous (regarding their clinical, methodological, and statistical aspects). Meta-analysis results are presented graphically in forest plots. Heterogeneity between effect sizes of included studies was assessed by visual inspection of forest plots and by using the Chi-square test for heterogeneity between studies, which was expressed as the percentage of the variability in effect estimates that is due to heterogeneity rather than chance (*I*^2^). In the meta-analysis, the effect estimates were included as originally reported. When results were reported at several time points, the last follow-up was chosen for the meta-analysis. When results were reported for outcomes both as ‘any’ (e.g. any asthma or any food reaction) and allergic or IgE associated (e.g. allergic asthma or IgE-associated food allergy), the outcomes associated with allergy or IgE associated were chosen. The meta-analysis was performed using Stata 14 (Stata Statistical Software: Release 14. College Station, TX: StataCorp LP).

Strength of evidence was appraised based on risk of bias, consistency/heterogeneity and precision of the evidence, according to the World Cancer Research Fund’s grading: ‘Convincing’, ‘Probable’, ‘Limited – suggestive’, ‘Limited – no conclusion’, and ‘Substantial effects unlikely’ (26).

## Results

**Fig. 1:**
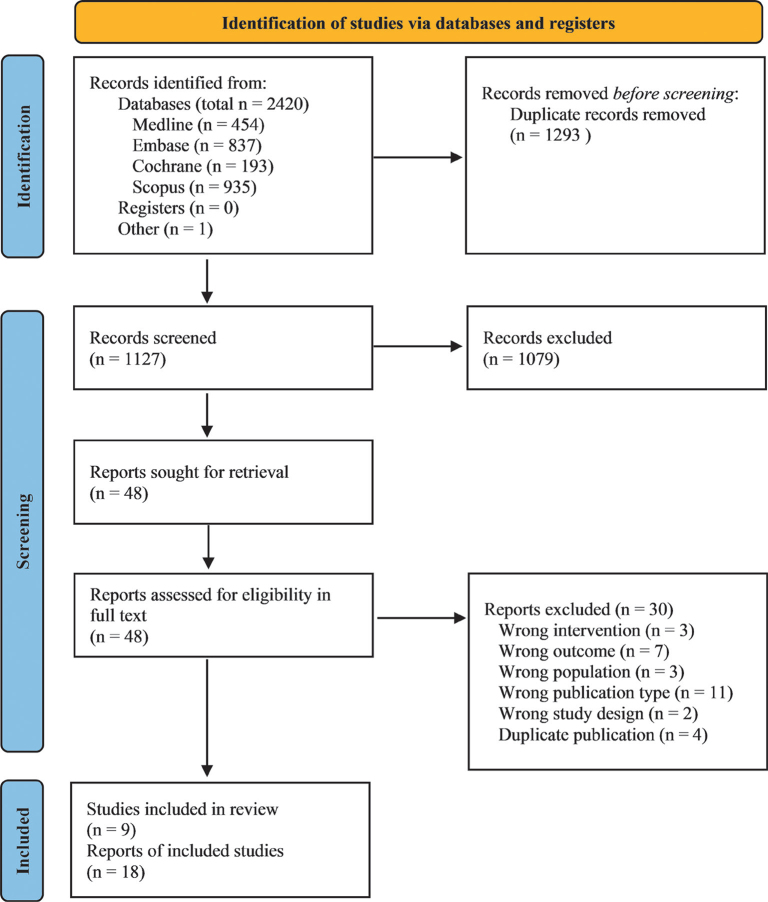
Prisma flow chart of the article selection process.

A total of 1,127 unique articles were identified and 48 read in full text after screening of title and abstract (Figure 1). Excluded articles with reasons is shown in Supplement 2. A total of 18 articles reporting from nine RCTs were included in this review (Table 2). In six studies, the LCn3PUFA intervention was given during pregnancy (29–38). In two studies, LCn3PUFA intervention was given during infancy and early childhood (39–44). In one additional study, LCn3PUFA intervention was given both during pregnancy and infancy as maternal supplementation continued during lactation (45, 46). In five of the studies (31, 35, 39, 40, 45, 47), only infants or women carrying children with high risk of developing atopic disease due to heredity were included.

**Table 2:** Description of the included studies

Author, year (ref) *Country*	Intervention	N recruited (% lost to follow-up)	Intervention, daily intake	Control	Follow-up at age
Furuhjelm 2009 (45) Furuhjelm 2011 (46) *Sweden*	Pregnancy and lactation (from GW 25)	*N* = 145 (19%) *N* = 145 (18%)	Capsules (4,500 mg) 35% EPA 25% DHA	Soy oil	1 years 2 years
Bisgaard 2016 (29) *Denmark*	Pregnancy (from GW 22–26)	*N* = 365 (5.6%)	Marine oil 2.4 g n-3 1.3 g EPA 0.9 g DHA	Olive oil	1–1.5, 3–5, 5 and 7 years
Olsen 2008 (30) *Denmark*	Pregnancy (from GW 30)	*N* = 402 (<1%)	Fish oil capsules 2.7 g n-3 0.9 g EPA 0.6 g DHA	Olive oil *(3*^rd^ *arm received nothing and are not included here)*	16 years
Dunstan 2003 (31) *Australia*	Pregnancy (from GW 20–30)	*N* = 98 (15%)	Fish oil capsules 3.7 g n-3 1.0 g EPA 2.1 g DHA	Olive oil	1 years
*DOMInO study* Palmer 2012 (32) Palmer 2013 (33) Best 2016 (34) Best 2018 (35) *Australia*	Pregnancy (from GW 21)	*N* = 706 (4%) *N* = 706 (10%) *N* = 706 (<1% in ITT) *N* = 706, cumulative analysis of previous studies	Fish oil 0.9 g n-3 0.1 g EPA 0.8 g DHA	Rapeseed, sunflower, and palm oil blend (1,500 mg/d)	1 years 3 years 6 years 1–6 years
Imhoff-Kunsch 2011 (36)Escamilla-Nunez 2014 (37) *Mexico*	Pregnancy (from GW 18–22)	*N* = 1,094 (22%) *N* = 1,094 (?%)	Algae derived DHA 0.4 g DHA	Corn and soy oil blend	1–6 months 1.5 years
Berman 2016 (38) *USA*	Pregnancy (from GW 12–20)	*N* = 126 (33%)	1) EPA-rich fish oil (1.06 g EPA and 0.274 g DHA) 2) DHA rich fish oil (0.9 g DHA and 0.180 g EPA)	Soy oil	3 years
*CAPS study* Mihrshahi 2003 (40) Peat 2004 (41) Marks 2006 (42) Toelle 2010 (43) Toelle 2013 (44) *Australia*	Infancy (6 months–5 years)	*N* = 616 (12%) *N* = 616 (15%) *N* = 616 (16%) *N* = 616 (27%) *N* = 616 (40%)	Tuna fish oil (500 mg) plus canola-based oils and spreads)	Sunola oil supplements plus oils and spreads low in n-3 fatty acids	1.5 years 3 years 5 years 8 years 11.5 years
D’Vaz 2012 (39) *Australia*	Infancy (birth until 6 months)	*N* = 420 (23%)	Fish oil (650 mg) 0.11 g EPA 0.28 g DHA	Olive oil	1 years

GW: gestational week; n-3: omega 3; EPA: eicosapentaenoic acid; DHA: docosahexaenoic acid.

### Interventions during pregnancy

In the Swedish study conducted during both pregnancy and during lactation (45, 46), Furuhjelm et al. randomized pregnant women to LCn3PUFA supplementation or soy oil placebo that continued throughout lactation. Among the six studies where the LCn3PUFA intervention was performed only during pregnancy, two were conducted in Denmark (29, 30), two in Australia (31–34), one in Mexico (36, 37), and one in the US (38). Interventions started in mid-pregnancy and continued throughout pregnancy. Doses of LCn3PUFA supplemental intake (either total n-3 or calculated sum of EPA and DHA) ranged from 0.4 to 3.7 g/day, DHA from 0.4 to 2.1 g/day, and EPA from 0 to 1.5 g/day. Placebo controls were vegetable oils (olive, soy, or blends) (Table 2).

#### Long chain n-3 fatty acid supplementation for the prevention of asthma or wheeze

**Fig. 2:**
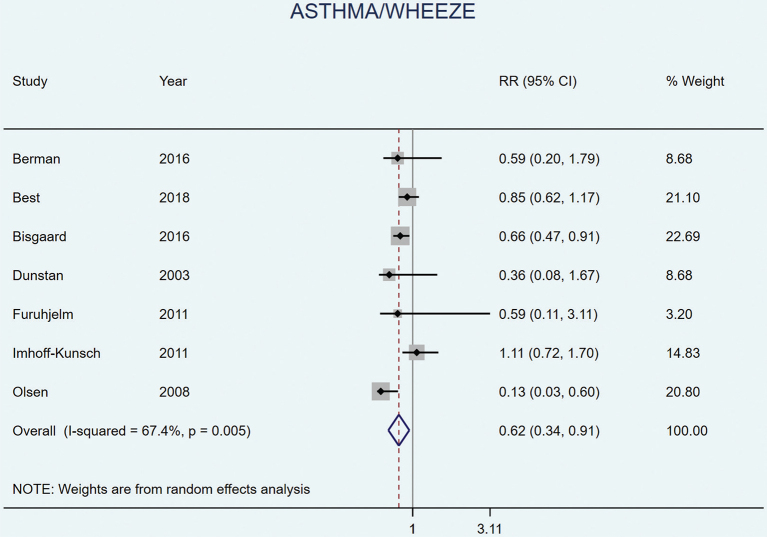
Random effects meta-analyses of long chain omega 3 supplementation during pregnancy on the risk of offspring asthma and/or wheeze.

Seven studies included asthma and/or wheeze as an outcome. The two studies conducted in Denmark (29, 30) both found a significant, protective effect of LCn3PUFA supplementation during pregnancy for asthma and/or persistent wheeze (Table 3). Bisgaard et al. found a ~30% reduced risk of asthma and/or persistent wheeze at 3–5 years of age, which persisted at 5 years and 5–7 years (29). Subgroup analyses showed that the effect was mainly seen among women with low EPA and DHA levels in blood at baseline (29). Olsen et al. found a ~60% reduced risk for any asthma and ~90% reduced risk of allergic asthma at 16 years of follow-up. Olsen et al. also found a non-significant protective effect for any asthma in the third arm receiving no oil compared to olive oil (HR [95% CI] 0.29 [0.08–1.03]) (30). The other individual studies found no significant effects on asthma and/or wheeze (31, 35, 38, 46). Meta-analysis of all seven studies (29–31, 35, 36, 38, 46) showed a reduction in the incidence of asthma or wheeze in the child from LCn3PUFA supplementation during pregnancy corresponding to RR 0.62 (95% CI 0.34–0.91, *P* = 0.005, *I*^2^ = 67.4%) (Figure 2). No dose–response analysis was conducted due to the limited number of studies.

**Table 3:** Results for long chain n-3 supplementation interventions conducted during pregnancy

Author, year (ref)*Country*	Asthma and/or wheeze	Eczema	Food allergy	Sensitization	Other outcomes
Furuhjelm 2009 (45) Furuhjelm 2011 (46) *Sweden*	IgE-associated asthma: RR (95% CI)*: 1 year: N/A 0–2 years: 0.59 (0.11–3.11) Any asthma: RR (95% CI)*: 1 year: N/A 0–2 years: 1.05 (0.41–2.72)	OR (95% CI): 1 year: 0.22 (0.06–0.81) 0–2 years: 0.33 (0.1–1.1)	OR (95% CI): 1 year: 0.09 (0.01–0.74) 0–2 years: 0.26 (0.07–0.99)	Any skin prick test OR (95% CI): 1 year: 0.36 (0.14–0.95) 0–2 years: 0.43 (0.17–1.10) IgE RR (95% CI)*: 1 year: 0.53 (0.24–1.20) 0–2 years: 0.75 (0.39–1.45)	Any allergic disease OR (95% CI): 1 year: N/A 0–2 years: 0.29 (0.1–0.86)
Bisgaard 2016 (29) *Denmark*	Persistent wheeze or asthma: HR (95% CI): 0–3 or 5 years: 0.69 (0.49–0.97) 0–5 years: 0.68 (0.49–0.95) 0–7 years: 0.66 (0.47–0.91)	HR (95% CI): 0–3 or 5 years: 1.19 (0.89–1.57) 0–5 years: 1.10 (0.83–1.44)	N/A	Any skin prick test OR (95% CI): 0.5–1.5 years: 1.34 (0.76–2.37) IgE OR (95% CI): 0.5–1.5 years: 1.72 (0.96–3.15)	Allergic rhinoconjunctivitis OR (95% CI): 0–5 years: 0.70 (0.43–1.12) Lung function tests 0–5 years: all NS
Olsen 2008 (30) *Denmark*	Allergic asthma 0–16 years HR (95% CI): 0.13 (0.03–0.60) Any asthma 0–16 years HR (95% CI): 0.37 (0.15–0.92)	N/A	N/A	N/A	Allergic asthma, atopic dermatitis, or allergic rhinitis 0–16 years HR (95% CI): 0.31 (0.11–0.84)
Dunstan 2003 (31) *Australia*	Asthma 1 year RR (95% CI)*: 0.36 (0.08–1.67)	Atopic dermatitis 1 year RR (95% CI)*: 1.49 (0.84–2.63)	Food allergy 1 year RR (95% CI)*: 0.65 (0.17–2.53)	Any skin prick test 1 year RR (95% CI)*: 0.68 (0.34–1.37)	N/A
*DOMInO* Palmer 2012 (32) Palmer 2013 (33) Best 2016 (34) Best 2018 (35) *Australia*	Asthma/wheeze with sensitization RR (95% CI) 1 year: N/A 1–3 years*: 1.10 (0.34 to 3.58) 6 years: N/A 0–6 years: 0.85 (0.62–1.17)	Eczema with sensitization RR (95% CI) 1 year: 0.64 (0.40 to 1.03) 1–3 years: 0.75 (0.53–1.05) 6 years: 0.95 (0.59–1.53) 0–6 years: 0.77 (0.53–1.13)	Food allergy with sensitization RR (95% CI) 1 year: 0.96 (0.41 to 2.25) 1–3 years: 1.25 (0.63–2.49) 6 years: N/A 0–6 years: N/A	Sensitization RR (95% CI) 1 year: 0.75 (0.53 to 1.04) 1–3 years: 0.85 (0.68–1.06) 6 years: 0–6 years: 0.97 (0.82–1.15)	Allergic disease with sensitization RR (95% CI) 1 year: 0.70 (0.45 to 1.09) 1–3 years: 0.78 (0.58–1.06) 6 years: 1.04 (0.82–1.33) 0–6 years: 0.88 (0.69, 1.12) Allergic rhinitis with sensitization RR (95% CI) 1 year: N/A 1–3 years: 0.82 (0.43–1.53) 6 years: 0.98 (0.72–1.35) 0–6 years: 0.86 (0.63, 1.16) Rhino-conjunctivitis with sensitization: RR (95% CI) 1 year: N/A 1–3 years: N/A 6 years: 1.12 (0.72–1.73) 0–6 years: 0.81 (0.55, 1.21)
Imhoff-Kunsch 2011 (36) Escamilla-Nunez 2014 (37) *Mexico*	Wheezing OR (95% CI) 0.5 year: 1.11 (0.72–1.70) IRR (95% CI) 1.5 years, maternal atopy: 0.88 (0.64–1.21) 1.5 years, non-maternal atopy: 1.03 (0.83–1.28)	N/A	N/A	N/A	N/A
Berman (38) *USA*	Asthma/wheeze 3 years RR (95% CI)* DHA: 0.59 (0.20–1.79) EPA: 1.12 (0.48–2.60)	Eczema 3 years OR (95% CI) DHA: 9.5 (1.5–59.6) EPA: 8.1 (1.4–46.3)	Food allergy 3 years RR (95% CI)* 1.73 (0.46–6.52)	N/A	N/A

* Calculated from reported n of events.

IgE: immunoglobulin E; RR: risk ratio; CI: confidence interval; y: years; OR: odds ratio; HR: hazard ratio; n-3: omega 3; EPA: eicosapentaenoic acid; DHA: docosahexaenoic acid.

#### Long chain n-3 supplementation for the prevention of eczema or atopic dermatitis

**Fig. 3:**
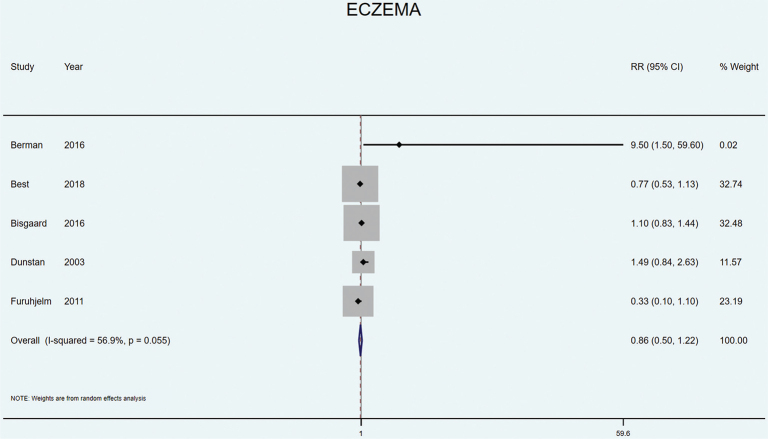
Random effects meta-analyses of long chain omega 3 supplementation during pregnancy on the risk of offspring eczema or atopic dermatitis.

Five studies included eczema or atopic dermatitis as an outcome. Furuhjelm et al. found that LCn3PUFA supplementation resulted in reduced risk of IgE-associated eczema at both 1 year (45) and during the first 2 years of childhood (*P* = 0.06 in adjusted analyses) (46). In contrast, Berman et al. found a significantly higher prevalence of eczema from LCn3PUFA supplements rich in either EPA or DHA (38). No other study found any effect of LCn3PUFA supplementation on the risk of eczema in adjusted analyses (29, 31, 35). Meta-analysis of all five studies (29, 31, 35, 38, 46) showed no significant effect of LCn3PUFA supplementation during pregnancy for eczema/atopic dermatitis in the child (RR 0.86 [95% CI 0.50–1.22], *P* = 0.055, *I*^2^ = 56.9%) (Figure 3).

#### Long chain n-3 fatty acid supplementation for the prevention of food allergy

**Fig. 4:**
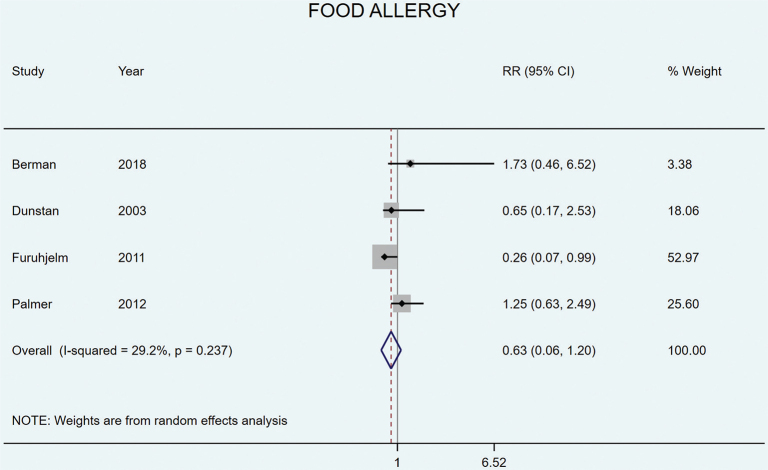
Random effects meta-analyses of long chain omega 3 supplementation during pregnancy on the risk of offspring food allergy.

Four studies included food allergy as an outcome. Furuhjelm et al. found that LCn3PUFA supplementation resulted in reduced risk of food allergy at both 1 year (45) and during the first 2 years of childhood (46). None of the other studies found any effects from LCn3PUFA supplementation on food allergy (31, 33, 38). Meta-analysis of all four studies (31, 33, 38, 46) showed no significant effect from LCn3PUFA supplementation on food allergy (RR 0.63 [95% CI 0.06–1.20], *P* = 0.237, *I*^2^ = 29.2%) (Figure 4).

#### Long chain n-3 fatty acid supplementation for the prevention of allergic sensitization

**Fig. 5:**
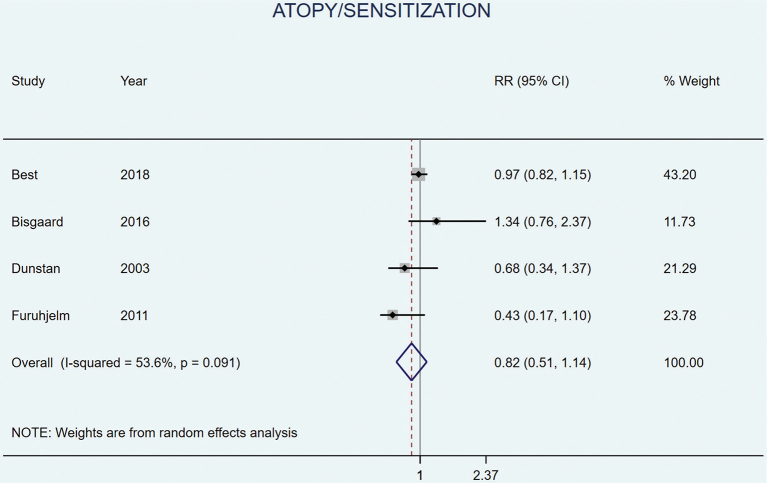
Random effects meta-analyses of long chain omega 3 supplementation during pregnancy on the risk of offspring atopy/sensitization.

Four studies included allergic sensitization or atopy (defined as positive skin prick test or IgE) as an outcome; none found any significant effects from LCn3PUFA supplementation in adjusted analyses (31, 35, 38, 46). Meta-analysis showed no significant effect on sensitization or atopy (RR 0.82 [95% CI 0.51–1.14], *P* = 0.091, *I*^2^ = 53.6%) (Figure 5).

#### Long chain n-3 fatty acid supplementation for the prevention of other atopic outcomes

No study found any significant effect from LCn3PUFA supplementation on allergic rhinoconjunctivitis (29, 35, 46) or allergic rhinitis (35). As <3 studies included these outcomes, no meta-analyses were conducted.

### Interventions during infancy

In two studies, the LCn3PUFA intervention was given during infancy; both conducted in Australia (39–44). D’Vaz et al. randomized 420 infants to receive either fish oil (650 mg/d, providing 280 mg DHA and 110 mg EPA) or olive oil control (650 mg/d) from birth to 6 months of age. When the children were 1 year old, LCn3PUFA supplementation showed no effect on incidence of allergic disease, eczema, food allergy, or sensitization (39) (Table 3). In the CAPS study, 616 infants were randomized to receive either tuna fish oil intervention (500 mg/d) or soy oil control from age 6 months (or at onset of bottle feeding) until 5 years. Half of the participants were also randomized to house dust mite avoidance. LCn3PUFA supplementation resulted in reduction incidence of wheeze at 1.5 years (40), but not at 3 years (41), 8 years (43), or 11.5 years (44). There were no reductions in incidence of asthma, eczema, atopy/sensitization, or rhinitis at any time point of follow-up (40–44).

**Table 4:** Results for long chain n-3 supplementation interventions conducted during infancy

Author, year (ref)*Country*	Asthma	Wheeze	Eczema	Food allergy	Sensitization	Other outcomes
*CAPS study* Mihrshahi 2003 (40) Peat 2004 (41) Marks 2006 (42) Toelle 2010 (43) Toelle 2013 (44) *Australia*	Abs RR (95% CI): 1.5 years: 3 years: NS 5 years: 0.9 (0.65–1.24) 8 years: –4.8 (–12.5–2.9) 11.5 years: 0.5 (–8.0–8.9)	Abs RR (95% CI): 1.5 years: 9.8 (1.5–18.1)% difference in events 3 years: NS 5 years: 8 years: –8.6 (16.8–0.4) 11.5 years: –1.1 (–9.9–7.8)	Abs RR (95% CI): 1.5 years: 0.6 (–5.7–6.9)% difference in events 3 years: NS 5 years: 1.39 (1.0–1.93) 8 years: –1.1 (–7.8–5.6) 11.5 years: 2.8 (–5.9–11.3)	Abs RR (95% CI): 1.5 years: 3 years: 1.6% (NS) 5 years: 8 years: 11.5 years:	Abs RR (95% CI): 1.5 years: 2.9 (–3.9–9.5)% difference in events 3 years: 5 years: 0.9 (0.74–1.1) 8 years: –0.2 (–9.9–9.6) 11.5 years: 12.4 (1.0–23.7)	Rhinitis Abs RR (95% CI): 1.5 years: 3 years: 5 years: 1.08 (0.88–1.33) 8 years: –0.9 (–9.4–7.6) 11.5 years: 7.3 (–2.6–17.2)
D’Vaz 2012 (39) *Australia*	N/A	OR (95% CI): 1 year: 1.51 (0.75–3.04)*	OR (95% CI): 1 year: 0.95 (0.6–1.5)*	OR (95% CI): 1 year: 0.78 (0.41.5)*	OR (95% CI): 1 year: 1.01 (0.62–1.65)*	Any allergic disease OR (95% CI): 1 year: 0.94 (0.60–1.47)*

* Estimated from graph.

Abs RR: absolute risk ratio; CI: confidence interval; y: years.

### Study quality and strength of evidence

**Fig. 6:**
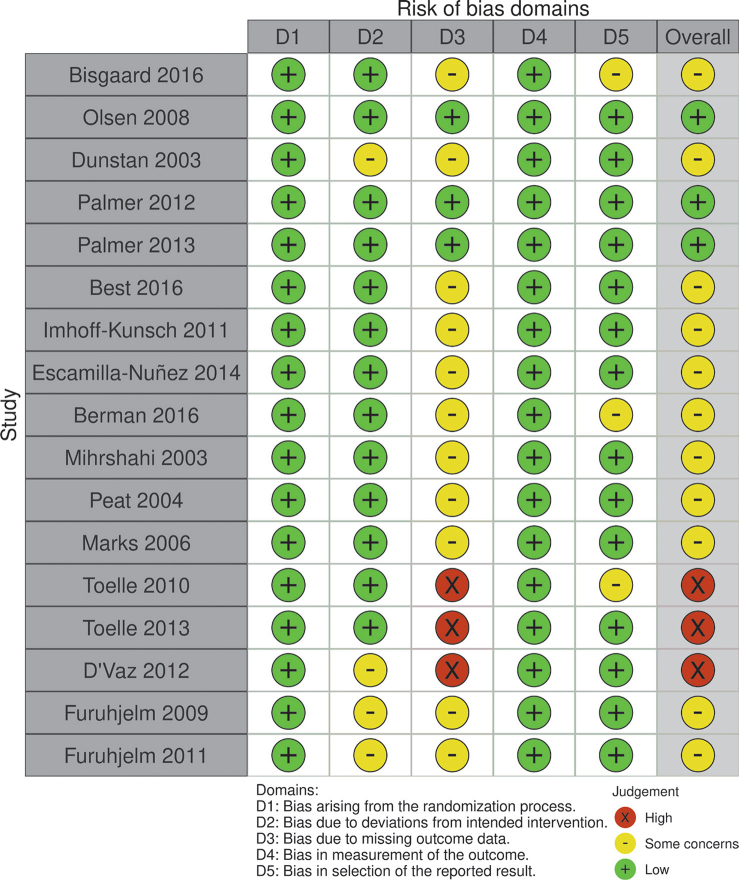
Traffic plot of the risk of bias assessment for each study, per domain and overall.

**Fig. 7:**
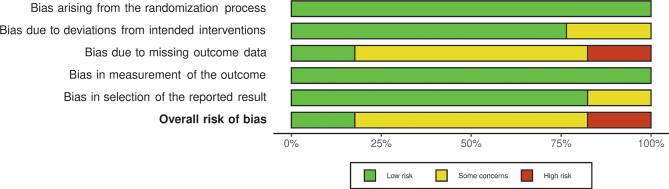
Summary plot of the risk of bias assessment, per domain and overall.

For interventions performed during pregnancy, risk of bias was considered as *low* for two studies (30, 32, 33), while most studies were considered to have *some concerns* regarding risk of bias (29, 31, 34, 36–38, 45, 46) (Figures 6 and 7). Risk of bias assessment for Best et al. 2018 (35) was not conducted as it did not include any new follow-up, only a cumulative summary of the previous papers that were assessed for risk of bias (32–34). For interventions during infancy, risk of bias was considered high for the IFOS study, due to concerns regarding missing outcome data (39). For the CAPS study, risk of bias was considered as having *some concerns* for the earlier follow-ups (40–42) and *high* for the later follow-ups (43, 44).

Strength of evidence that LCn3PUFA supplementation during pregnancy reduces risk of asthma and/or wheeze in the offspring was considered *Limited-suggestive (low)*, due to risk of bias, some unexplained heterogeneity in results, definitions of outcomes, and given interventions. Evidence that the LCn3PUFA supplementation during pregnancy reduces risk of eczema, food allergy, allergic sensitization, or other atopic outcomes in the offspring was considered *Limited-inconclusive (low)* due to large unexplained heterogeneity in results and low precision in the effect estimates. Strength of evidence that LCn3PUFA supplementation during infancy (either given directly to the infant or to the lactating mother) reduces risk of asthma/wheeze, eczema, allergy, or atopy was considered *Limited – no conclusion (insufficient)*.

## Discussion

The results of this systematic review of RCTs show that LCn3PUFA supplementation during pregnancy might reduce the risk of asthma and/or wheeze in the offspring, but evidence for other atopic conditions was insufficient. Supplementation during infancy did not yield any clear effects.

The current results are comparable to those of previous systematic reviews, concluding that fish oil or LCn3PUFA supplementation given during pregnancy, lactation, or in infancy may not reduce allergy or atopic disease (48–50). We did, however, find low-quality evidence for a protective effect for childhood asthma and/or wheeze, in line with findings from other recent systematic reviews (16, 51, 52). Earlier reviews finding no such effects were published in 2014–2016 after which new data have been published (29, 34, 38). Still, the strength of evidence is considered low, due to study heterogeneity and risk of bias.

The results of this systematic review show that the suggestive protective effects of LCn3PUFA supplementation during pregnancy are limited to asthma and/or wheeze and not other atopic conditions. In the COPSAC study, a significantly reduced risk of asthma and/or recurrent wheeze from LCn3PUFA supplementation was found, but no effect on lung function or asthma exacerbations. However, LCn3PUFA supplementation reduced the risk of lower respiratory tract infections (29), an outcome outside the scope of this systematic review. The here included study conducted in Mexico also found lower risk of symptoms of common cold at 1 month and until 18 months of age (36, 37). It is possible that LCn3PUFA supplementation reduces risk of asthma/wheeze through a reduction in risk of respiratory infections that may both induce and exacerbate asthma (48). It is noteworthy that most studies were conducted among children with a high risk of developing atopic disease, and studies in the general population may result in different findings. In addition, definitions of the outcome asthma and/or wheeze differed between studies and included a combination of asthma, allergic asthma, and wheeze. Classification of asthma and/or wheeze was performed using different assessments such as clinical diagnosis, questionnaires and symptoms diaries, and at different ages. Initially, we planned to only include outcomes based on doctors’ diagnosis, but since very few studies applied this criterion, and diagnosis of certain outcomes (e.g. asthma) in small children is difficult, this was discarded. Taken together, more RCT studies are required that use clearly defined endpoints and valid assessments to clarify the role of LCn3PUFA supplementation during pregnancy for the prevention of childhood asthma.

This systematic review has both strengths and limitations. Strengths include the methodology with two separate co-authors doing article selection, data extraction, and risk of bias assessment. There was also a fair amount of data for both meta-analyses and evidence synthesis. However, the number of included studies were too few to conduct analyses of publication bias and for subgroup or sensitivity analyses. Limitations include heterogeneity in both interventions, follow-up, and in reporting of data in the original studies and, therefore, also in the current paper (e.g. doses, follow-up duration, and classification of outcomes).

In conclusion, the intake of LCn3PUFA supplements during pregnancy may reduce the risk of asthma and/or wheeze in the offspring, but the strength of evidence is low. There is inconclusive evidence for the effects of LCn3PUFA supplements during pregnancy for other atopic outcomes as well as for supplementation during lactation or infancy.

## Supplementary Material

Supplementation with long chain n-3 fatty acids during pregnancy, lactation, or infancy in relation to risk of asthma and atopic disease during childhood: a systematic review and meta-analysis of randomized controlled clinical trials
