# Supplementary material for: Supplementation with long chain n-3 fatty acids during pregnancy, lactation, or infancy in relation to risk of asthma and atopic disease during childhood: a systematic review and meta-analysis of randomized controlled clinical trials
Source: Food Nutr Res. 2022 Oct 11;66:10.29219/fnr.v66.8842. doi: 10.29219/fnr.v66.8842 (PMC9602204; doi:10.29219/fnr.v66.8842)
Supplement: Supplementation with long chain n-3 fatty acids during pregnancy, lactation, or infancy in relation to risk of asthma and atopic disease during childhood: a systematic review and meta-analysis of randomized controlled clinical trials [file FNR-66-8842-s001.zip › Supplement 1..docx]

Documentation of search strategies

University Library search consultation group

Date: 20 September 2021

Topic/research question: Supplementation with long chain n3 fatty acids during pregnancy, lactation or infancy in relation to risk of asthma and allergy during childhood

Name of researcher(s): Agneta Åkesson & Linnea Bärebring

Librarian(s): Sabina Gillsund & Narcisa Hannerz

Databases:

1. Medline (Ovid)
2. Embase (embase.com)
3. Cochrane (Wiley
4. Scopus (Elsevier)

Total number of hits:

- Before deduplication: 2,419
- After deduplication: 1,126

Comments:

1. Medline

| Interface: Ovid MEDLINE(R) and Epub Ahead of Print, In-Process & Other Non-Indexed Citations and Daily  Date of Search: 20 September 2021  Number of hits: 454  Comment: In Ovid, two or more words are automatically searched as phrases; i.e. no quotation marks are needed | Field labels   - exp/ = exploded MeSH term - / = non exploded MeSH term - .ti,ab,kf. = title, abstract and author keywords - adjx = within x words, regardless of order - * = truncation of word for alternate endings |
| --- | --- |
| Database(s): **Ovid MEDLINE(R) and Epub Ahead of Print, In-Process, In-Data-Review & Other Non-Indexed Citations and Daily**1946 to September 20, 2021 Search Strategy:   \| **#** \| **Searches** \| **Results** \| \| --- \| --- \| --- \| \| 1 \| exp Pregnancy/ \| 935377 \| \| 2 \| Pregnant Women/ \| 10432 \| \| 3 \| exp Pregnancy Trimesters/ \| 42588 \| \| 4 \| Peripartum Period/ \| 1428 \| \| 5 \| Fetus/ \| 79997 \| \| 6 \| Postpartum Period/ \| 27551 \| \| 7 \| exp Lactation/ \| 44576 \| \| 8 \| exp Breast Feeding/ \| 40316 \| \| 9 \| Milk, Human/ \| 20644 \| \| 10 \| exp Infant/ \| 1186920 \| \| 11 \| exp Maternal Nutritional Physiological Phenomena/ \| 6605 \| \| 12 \| Infant Nutritional Physiological Phenomena/ \| 15687 \| \| 13 \| (antenatal* or ante natal* or birth* or child bearing or childbearing or childbirth* or fetal or fetus* or foetal or foetus* or gestation* or maternal* or perinatal* or peri natal* or peripartum or peri partum or postpartum or post partum or prematur* or preterm or prenatal* or pre natal* or pregnan* or puerperium).ti,ab,kf. \| 1346831 \| \| 14 \| (baby or babies or infant* or infancy or neonat* or newborn* or offspring).ti,ab,kf. \| 896151 \| \| 15 \| (breast feed* or breastfeed* or breastfed or breast fed or breast milk or breastmilk or breast pump* or breastpump* or human milk or lactation* or lactating).ti,ab,kf. \| 112578 \| \| 16 \| or/1-15 \| 2727007 \| \| 17 \| exp Fish Oils/ \| 29861 \| \| 18 \| (fatty acid* adj3 (long chain or n3 or n-3)).ti,ab,kf. \| 23787 \| \| 19 \| (docosahexaenoic acid* or docosahexaenoate or eicosapentaenoic acid* or icosapent or omega-3 or omega3 or timnodonic acid*).ti,ab,kf. \| 30807 \| \| 20 \| ((n3 or n-3) adj3 (marine or oil* or pufa*)).ti,ab,kf. \| 6535 \| \| 21 \| ((cod or codfish or codliver or fish or marine or tuna) adj3 oil*).ti,ab,kf. \| 13958 \| \| 22 \| or/17-21 \| 59275 \| \| 23 \| exp Hypersensitivity/ \| 358457 \| \| 24 \| Rhinitis/ \| 13500 \| \| 25 \| Conjunctivitis/ \| 6256 \| \| 26 \| exp Eczema/ \| 11786 \| \| 27 \| exp Immunoglobulin E/ \| 42882 \| \| 28 \| (airway hyper-responsiveness or allerg* or anaphyla* or angioedema* or angioneurotic edema* or angioneurotic oedema* or asthma* or atop* or besnier* prurigo or conjunctiviti* or dermatiti* or eczema* or exercise-induced bronchospasm* or hayfever* or hay fever* or hives or hypersensitivit* or "immunoglobulin e" or IgE or nasal catarrh* or neurodermatiti* or pollen or pollenosis or pollinosis or quincke* edema* or quincke* oedema* or respiratory hyper-responsiveness or rhinit* or rhinoconjunctiviti* or urticari* or vernal keratoconjunctivitis* or wheez*).ti,ab,kf. \| 523542 \| \| 29 \| or/23-28 \| 616571 \| \| 30 \| 16 and 22 and 29 \| 472 \| \| 31 \| (docosahexaenoic acid* or docosahexaenoate or eicosapentaenoic acid* or icosapent or omega-3 or omega3 or timnodonic acid*).ti,ab,kf. \| 30807 \| \| 32 \| 22 or 31 \| 59275 \| \| 33 \| 16 and 29 and 32 \| 472 \| \| 34 \| 33 not (animals not humans).sh. \| 454 \| | |

2. Embase

| Interface: embase.com  Date of Search: 20 September 2021  Number of hits: 837  Comment: Emtree is the controlled vocabulary in Embase | Field labels   - /exp = exploded Emtree term - /de = non exploded Emtree term - ti,ab,kw = title, abstract and author keywords - NEAR/x = within x words, regardless of order - * = truncation of word for alternate endings |
| --- | --- |
| **No.**  **Query**  **Results**  **837**  **#35**  **#33** NOT **#34**  **6,027,564**  **#34**  [animals]/lim NOT [humans]/lim  **865**  **#33**  **#15** AND **#25** AND **#32**  **975,302**  **#32**  **#26** OR **#27** OR **#28** OR **#29** OR **#30** OR **#31**  **729,629**  **#31**  **'airway hyper-responsiveness'**:ti,ab,kw OR **allerg***:ti,ab,kw OR **anaphyla***:ti,ab,kw OR **angioedema***:ti,ab,kw OR **'angioneurotic edema*'**:ti,ab,kw OR **'angioneurotic oedema*'**:ti,ab,kw OR **asthma***:ti,ab,kw OR **atop***:ti,ab,kw OR **'besnier* prurigo'**:ti,ab,kw OR **conjunctiviti***:ti,ab,kw OR **dermatiti***:ti,ab,kw OR **eczema***:ti,ab,kw OR **'exercise-induced bronchospasm*'**:ti,ab,kw OR **hayfever***:ti,ab,kw OR **'hay fever*'**:ti,ab,kw OR **hives**:ti,ab,kw OR **hypersensitivit***:ti,ab,kw OR **'immunoglobulin e'**:ti,ab,kw OR **ige**:ti,ab,kw OR **'nasal catarrh*'**:ti,ab,kw OR **neurodermatiti***:ti,ab,kw OR **pollen**:ti,ab,kw OR **pollenosis**:ti,ab,kw OR **pollinosis**:ti,ab,kw OR **'quincke* edema*'**:ti,ab,kw OR **'quincke* oedema*'**:ti,ab,kw OR **'respiratory hyper-responsiveness'**:ti,ab,kw OR **rhinit***:ti,ab,kw OR **rhinoconjunctiviti***:ti,ab,kw OR **urticari***:ti,ab,kw OR **'vernal keratoconjunctivitis*'**:ti,ab,kw OR **wheez***:ti,ab,kw  **88,018**  **#30**  **'immunoglobulin e'**/exp  **32,607**  **#29**  **'eczema'**/exp  **20,758**  **#28**  **'conjunctivitis'**/de  **21,080**  **#27**  **'rhinitis'**/de  **697,725**  **#26**  **'hypersensitivity'**/exp  **89,529**  **#25**  **#16** OR **#17** OR **#18** OR **#19** OR **#20** OR **#21** OR **#22** OR **#23** OR **#24**  **17,809**  **#24**  ((**cod** OR **codfish** OR **codliver** OR **fish** OR **marine** OR **tuna**) NEAR/3 **oil***):ti,ab,kw  **8,100**  **#23**  ((**n3** OR **'n 3'**) NEAR/3 (**marine** OR **oil*** OR **pufa***)):ti,ab,kw  **37,333**  **#22**  **'docosahexaenoic acid*'**:ti,ab,kw OR **docosahexaenoate**:ti,ab,kw OR **'eicosapentaenoic acid*'**:ti,ab,kw OR **icosapent**:ti,ab,kw OR **'omega 3'**:ti,ab,kw OR **omega3**:ti,ab,kw OR **'timnodonic acid*'**:ti,ab,kw  **28,815**  **#21**  (**'fatty acid*'** NEAR/3 (**'long chain'** OR **n3** OR **'n 3'**)):ti,ab,kw  **18,266**  **#20**  **'icosapentaenoic acid'**/exp  **22,988**  **#19**  **'docosahexaenoic acid'**/exp  **34,290**  **#18**  **'omega 3 fatty acid'**/exp  **1,316**  **#17**  **'cod liver oil'**/exp  **18,168**  **#16**  **'fish oil'**/exp  **2,913,814**  **#15**  **#1** OR **#2** OR **#3** OR **#4** OR **#5** OR **#6** OR **#7** OR **#8** OR **#9** OR **#10** OR **#11** OR **#12** OR **#13** OR **#14**  **132,439**  **#14**  **'breast feed*'**:ti,ab,kw OR **breastfeed***:ti,ab,kw OR **breastfed**:ti,ab,kw OR **'breast fed'**:ti,ab,kw OR **'breast milk'**:ti,ab,kw OR **breastmilk**:ti,ab,kw OR **'breast pump*'**:ti,ab,kw OR **breastpump***:ti,ab,kw OR **'human milk'**:ti,ab,kw OR **lactation***:ti,ab,kw OR **lactating**:ti,ab,kw  **1,090,619**  **#13**  **baby**:ti,ab,kw OR **babies**:ti,ab,kw OR **infant***:ti,ab,kw OR **infancy**:ti,ab,kw OR **neonat***:ti,ab,kw OR **newborn***:ti,ab,kw OR **offspring**:ti,ab,kw  **1,711,976**  **#12**  **antenatal***:ti,ab,kw OR **'ante natal*'**:ti,ab,kw OR **birth***:ti,ab,kw OR **'child bearing'**:ti,ab,kw OR **childbearing**:ti,ab,kw OR **childbirth***:ti,ab,kw OR **fetal**:ti,ab,kw OR **fetus***:ti,ab,kw OR **foetal**:ti,ab,kw OR **foetus***:ti,ab,kw OR **gestation***:ti,ab,kw OR **maternal***:ti,ab,kw OR **perinatal***:ti,ab,kw OR **'peri natal*'**:ti,ab,kw OR **peripartum**:ti,ab,kw OR **'peri partum'**:ti,ab,kw OR **postpartum**:ti,ab,kw OR **'post partum'**:ti,ab,kw OR **prematur***:ti,ab,kw OR **preterm**:ti,ab,kw OR **prenatal***:ti,ab,kw OR **'pre natal*'**:ti,ab,kw OR **pregnan***:ti,ab,kw OR **puerperium**:ti,ab,kw  **103,429**  **#11**  **'infant nutrition'**/exp  **13,103**  **#10**  **'maternal nutrition'**/exp  **120,076**  **#9**  **'prematurity'**/exp  **1,195,233**  **#8**  **'infant'**/exp  **59,209**  **#7**  **'breast feeding'**/exp  **57,685**  **#6**  **'lactation'**/exp  **74,699**  **#5**  **'puerperium'**/exp  **213,865**  **#4**  **'fetus'**/exp  **37,285**  **#3**  **'perinatal period'**/exp  **91,401**  **#2**  **'pregnant woman'**/exp  **808,264**  **#1**  **'pregnancy'**/exp | |

3. Cochrane Library

| Interface: Wiley  Date of Search: 20 September 2021  Number of hits: 198 (193 in Cochrane Central Register of Controlled Trials) | Field labels   - ti,ab,kw = title, abstract and author keywords - NEAR/x = within x words, regardless of order - * = truncation of word for alternate endings |
| --- | --- |
| \| **ID** \| **Search** \| **Hits** \| \| --- \| --- \| --- \| \| #1 \| MeSH descriptor: [Pregnancy] explode all trees \| 23152 \| \| #2 \| MeSH descriptor: [Pregnant Women] this term only \| 367 \| \| #3 \| MeSH descriptor: [Pregnancy Trimesters] explode all trees \| 1787 \| \| #4 \| MeSH descriptor: [Peripartum Period] this term only \| 16 \| \| #5 \| MeSH descriptor: [Fetus] this term only \| 344 \| \| #6 \| MeSH descriptor: [Postpartum Period] this term only \| 1274 \| \| #7 \| MeSH descriptor: [Lactation] explode all trees \| 640 \| \| #8 \| MeSH descriptor: [Breast Feeding] explode all trees \| 1977 \| \| #9 \| MeSH descriptor: [Milk, Human] this term only \| 1062 \| \| #10 \| MeSH descriptor: [Infant] explode all trees \| 33346 \| \| #11 \| MeSH descriptor: [Maternal Nutritional Physiological Phenomena] explode all trees \| 457 \| \| #12 \| MeSH descriptor: [Infant Nutritional Physiological Phenomena] this term only \| 1077 \| \| #13 \| (antenatal* OR "ante natal*" OR birth* OR "child bearing" OR childbearing OR childbirth* OR fetal OR fetus* OR foetal OR foetus* OR gestation* OR maternal* OR perinatal* OR "peri natal*" OR peripartum OR "peri partum" OR postpartum OR "post partum" OR prematur* OR preterm OR prenatal* OR (pre NEXT natal*) OR pregnan* OR puerperium):ti,ab,kw \| 117537 \| \| #14 \| (baby OR babies OR infant* OR infancy OR neonat* OR newborn* OR offspring):ti,ab,kw \| 82221 \| \| #15 \| ((breast NEXT feed*) OR breastfeed* OR breastfed OR "breast fed" OR "breast milk" OR breastmilk OR (breast NEXT pump*) OR breastpump* OR "human milk" OR lactation* OR lactating):ti,ab,kw \| 14324 \| \| #16 \| #1 or #2 or #3 or #4 or #5 or #6 or #7 or #8 or #9 or #10 or #11 or #12 or #13 or #14 or #15 \| 157904 \| \| #17 \| MeSH descriptor: [Fish Oils] explode all trees \| 3585 \| \| #18 \| ((fatty NEXT acid*) NEAR/3 ("long chain" OR n3 OR "n-3")) \| 3060 \| \| #19 \| ((docosahexaenoic NEXT acid*) OR docosahexaenoate OR (eicosapentaenoic NEXT acid*) OR icosapent OR "omega-3" OR omega3 OR (timnodonic NEXT acid*)):ti,ab,kw \| 8316 \| \| #20 \| ((n3 or "n-3") NEAR/3 (marine or oil* or pufa*)):ti,ab,kw \| 1330 \| \| #21 \| ((cod OR codfish OR codliver OR fish OR marine OR tuna) NEAR/3 oil*):ti,ab,kw \| 3505 \| \| #22 \| #17 or #18 or #19 or #20 or #21 \| 10525 \| \| #23 \| MeSH descriptor: [Hypersensitivity] explode all trees \| 21381 \| \| #24 \| MeSH descriptor: [Rhinitis] this term only \| 1170 \| \| #25 \| MeSH descriptor: [Conjunctivitis] this term only \| 396 \| \| #26 \| MeSH descriptor: [Eczema] explode all trees \| 1125 \| \| #27 \| MeSH descriptor: [Immunoglobulin E] explode all trees \| 1348 \| \| #28 \| ("airway hyper-responsiveness" OR allerg* OR anaphyla* OR angioedema* OR (angioneurotic NEXT edema*) OR (angioneurotic NEXT oedema*) OR asthma* OR atop* OR (besnier* NEXT prurigo) OR conjunctiviti* OR dermatiti* OR eczema* OR ("exercise-induced" NEXT bronchospasm*) OR hayfever* OR (hay NEXT fever*) OR hives OR hypersensitivit* OR "immunoglobulin e" OR IgE OR (nasal NEXT catarrh*) OR neurodermatiti* OR pollen OR pollenosis OR pollinosis OR (quincke* NEXT edema*) OR (quincke* NEXT oedema*) OR "respiratory hyper-responsiveness" OR rhinit* OR rhinoconjunctiviti* OR urticari* OR (vernal NEXT keratoconjunctivitis*) OR wheez*):ti,ab,kw \| 79194 \| \| #29 \| #23 or #24 or #25 or #26 or #27 or #28 \| 79731 \| \| #30 \| #16 and #22 and #29 \| 198 \| | |

4. Scopus

| Interface: Elsevier  Date of Search: 20 September 2021  Number of hits: 935 | Field labels   - TITLE-ABS-KEY = title, abstract and author keywords - W/x = within x words, regardless of order - = truncation of word for alternate endings |
| --- | --- |
| ( ( TITLE-ABS-KEY ( "antenatal*" OR "ante natal*" OR birth* OR "child bearing" OR "childbearing" OR childbirth* OR "fetal" OR fetus* OR "foetal" OR foetus* OR gestation* OR maternal* OR "perinatal*" OR "peri natal*" OR "peripartum" OR "peri partum" OR "postpartum" OR "post partum" OR prematur* OR preterm OR prenatal* OR "pre natal*" OR pregnan* OR "puerperium" ) ) OR ( TITLE-ABS-KEY ( "baby" OR "babies" OR infant* OR "infancy" OR neonat* OR newborn* OR "offspring" ) ) OR ( TITLE-ABS-KEY ( "breast feed*" OR breastfeed* OR "breastfed" OR "breast fed" OR "breast milk" OR "breastmilk" OR "breast pump*" OR breastpump* OR "human milk" OR lactation* OR "lactating" ) ) ) AND ( ( TITLE-ABS-KEY ( "fatty acid*" W/2 ( "long chain" OR "n3" OR "n-3" ) ) ) OR ( TITLE-ABS-KEY ( "docosahexaenoic acid*" OR "docosahexaenoate" OR "eicosapentaenoic acid*" OR "icosapent" OR "omega-3" OR "omega3" OR "timnodonic acid*") ) OR ( TITLE-ABS-KEY ( ( "n3" OR "n-3" ) W/2 ( "marine" OR oil* OR pufa* ) ) ) OR ( TITLE-ABS-KEY ( ( "cod" OR "codfish" OR "codliver" OR "fish" OR "marine" OR "tuna" ) W/2 oil* ) ) ) AND ( TITLE-ABS-KEY ( "airway hyper-responsiveness" OR allerg* OR anaphyla* OR angioedema* OR "angioneurotic edema*" OR "angioneurotic oedema*" OR asthma* OR atop* OR "besnier* prurigo" OR conjunctiviti* OR dermatiti* OR eczema* OR "exercise-induced bronchospasm*" OR hayfever* OR "hay fever*" OR "hives" OR hypersensitivit* OR "immunoglobulin e" OR "IgE" OR "nasal catarrh*" OR neurodermatiti* OR "pollen" OR "pollenosis" OR "pollinosis" OR "quincke* edema*" OR "quincke* oedema*" OR "respiratory hyper-responsiveness" OR rhinit* OR rhinoconjunctiviti* OR urticari* OR "vernal keratoconjunctivitis*" OR wheez* ) ) | |
