# Supplementary material for: Supplementation with long chain n-3 fatty acids during pregnancy, lactation, or infancy in relation to risk of asthma and atopic disease during childhood: a systematic review and meta-analysis of randomized controlled clinical trials
Source: Food Nutr Res. 2022 Oct 11;66:10.29219/fnr.v66.8842. doi: 10.29219/fnr.v66.8842 (PMC9602204; doi:10.29219/fnr.v66.8842)
Supplement: Supplementation with long chain n-3 fatty acids during pregnancy, lactation, or infancy in relation to risk of asthma and atopic disease during childhood: a systematic review and meta-analysis of randomized controlled clinical trials [file FNR-66-8842-s001.zip › Supplement 2..docx]

| **Decision** | **Reason** | **Article** |
| --- | --- | --- |
| Exclude | Wrong study design (wrong research question) | Childhood fish oil supplementation modifies associations between traffic related air pollution and allergic sensitisation. Environmental health: a global access science source. 2018 2018;17(1). |
| Exclude | Wrong publication type (protocol registration) | Actrn. Fish oil supplementation in pregnancy to reduce allergies in early childhood. http://wwwwhoint/trialsearch/Trial2aspx?TrialID=ACTRN12610000735055. 2010 2010. |
| Exclude | Duplicate article | Actrn. Six year follow up of children at high hereditary risk of allergy born to mothers participating in the DOMInO Trial. http://wwwwhoint/trialsearch/Trial2aspx?TrialID=ACTRN12615000498594. 2015 2015. |
| Exclude | Wrong publication type (abstract) | Barraza-Villarreal A, Escamilla-Núnez C, Hern, ez-Cadena L, Navarro E, Sly PD, et al. Prenatal environmental exposure and respiratory infection in childhood from Cuernavaca, Mexico. American Journal of Respiratory and Critical Care Medicine. 2011 2011;183(1). |
| Exclude | Wrong publication type (abstract) | Barraza-Villarreal A, Hern, ez L, Escamilla C, Sly P, Neufeld LM, et al. Supplementation with omega-3 fatty acids and dermatitis atopic in infants: a randomized controlled trial. American thoracic society international conference, may 15-20 2009, san diego. 2009 2009:A2872 [Poster #H59]. |
| Exclude | Wrong publication type (Commentary on other paper) | Best K, Makrides M. Possible protective effect of prenatal omega-3 long-chain polyunsaturated fatty acids supplementation on persistent wheeze and asthma in early childhood. Evidence Based Medicine. 06;22(3):104. |
| Exclude | Wrong publication type (abstract) | Best K, Makrides M, Sullivan T, Michael G, Declan K, James M, et al. Prenatal omega-3 supplementation and allergy in childhood. European journal of pediatrics. 2016 2016;175(11):1437‐. |
| Exclude | Duplicate article | Best K, Sullivan T, Gold M, Kennedy D, Martin J, Palmer D, et al. Six-year follow up of children at high hereditary risk of allergy, born to mothers supplemented with docosahexaenoic acid (DHA) in the domino trial. Journal of paediatrics and child health. 2015 2015;51:58‐. |
| Exclude | Wrong intervention (DHA+ARA) | Birch EE, Khoury JC, Berseth CL, Castaneda YS, Couch JM, Bean J, et al. The impact of early nutrition on incidence of allergic manifestations and common respiratory illnesses in children. Journal of Pediatrics. Jun;156(6):902-6.e1. |
| Exclude | Duplicate article (same as Bisgaard, in spanish) | Chercoles ER. Fish oil-derived fatty acids in pregnancy and wheeze and asthma in offspring. Acta Pediatrica Espanola. 2017 2017;75(5):81. |
| Exclude | Wrong publication type (protocol registration) | Ctri. Fish oil supplementation in pregnancy for preventing allergy in children. http://wwwwhoint/trialsearch/Trial2aspx?TrialID=CTRI/2020/01/023058. 2020 2020. |
| Exclude | Wrong publication type (abstract) | Garden F, Toelle B, Marks G. Outcomes of the childhood asthma prevention study (CAPS) at age 14 years. Respirology (carlton, vic). 2016 2016;21:108‐. |
| Exclude | Wrong population (preterm infants) | Gunaratne AW, Makrides M, Collins CT, Gibson RA, McPhee AJ, Sullivan TR, et al. Docosahexaenoic acid supplementation of preterm infants and parent-reported symptoms of allergic disease at 7 years corrected age: follow-up of a randomized controlled trial. American journal of clinical nutrition. 2019 2019;109(6):1600‐10. |
| Exclude | Wrong publication type (abstract) | Gunaratne AW, Makrides M, Sullivan TR, Gibson RA, Collins CT. Is allergic disease reduced at 7-years corrected age in preterm infants who received high-dose docosahexaenoic acid (DHA) supplementation? Journal of paediatrics and child health. 2016 2016;52:57‐. |
| Exclude | Wrong population (wrong age) | Hansen S, Strom M, Maslova E, Dahl R, Hoffmann HJ, Rytter D, et al. Fish oil supplementation during pregnancy and allergic respiratory disease in the adult offspring. Journal of Allergy & Clinical Immunology. 01;139(1):104-11.e. |
| Exclude | Wrong outcome | Hjelmso MH, Shah SA, Thorsen J, Rasmussen M, Vestergaard G, Mortensen MS, et al. Prenatal dietary supplements influence the infant airway microbiota in a randomized factorial clinical trial. Nature communications. 01-22;11(1):426. |
| Exclude | Wrong publication type (abstract) | Jin X, Li F, Liu B, Zhuang W, Strong P, Scalabrin D. Consumption of a follow-up formula containing docosahexaenoic acid, prebiotics, and beta-glucan reduced the incidence and duration of acute respiratory infections in 3-4 year old children. Allergy: european journal of allergy and clinical immunology. 2013 2013;68:445. |
| Exclude | Wrong study design (cohort analysis) | Kachroo P, Kelly RS, Mirzakhani H, Lee-Sarwar K, Chawes BL, Blighe K, et al. Fish oil supplementation during pregnancy is protective against asthma/wheeze in offspring. The Journal of Allergy & Clinical Immunology in Practice. 01;8(1):388-91.e2. |
| Exclude | Wrong population (preterm infants) | Manley BJ, Makrides M, Collins CT, McPhee AJ, Gibson RA, Ryan P, et al. High-dose docosahexaenoic acid supplementation of preterm infants: respiratory and allergy outcomes. Pediatrics. 2011 2011;128(1):e71‐7. |
| Exclude | Wrong intervention (FA levels) | Mihrshahi S, Peat JK, Webb K, Oddy W, Marks GB, Mellis CM. Effect of omega-3 fatty acid concentrations in plasma on symptoms of asthma at 18 months of age. Pediatric allergy and immunology. 2004 2004;15(6):517‐22. |
| Exclude | Duplicate article (same as Palmer) | Palmer DJ, Sullivan T, Gold MS, Prescott SL, Heddle R, Gibson RA, et al. Effect of n-3 polyunsaturated fatty acid supplementation in pregnancy on early childhood allergic disease: randomized controlled trial. Journal of paediatrics and child health. 2013 2013;49:56‐. |
| Exclude | Wrong outcome | Pastor N, Soler B, Mitmesser SH, Ferguson P, Lifschitz C. Infants fed docosahexaenoic acid- and arachidonic acid-supplemented formula have decreased incidence of bronchiolitis/bronchitis the first year of life. Clinical Pediatrics. Nov;45(9):850-5. |
| Exclude | Wrong outcome | Romero VC, Somers EC, Stolberg V, Clinton C, Chensue S, Djuric Z, et al. Developmental programming for allergy: a secondary analysis of the Mothers, Omega-3, and Mental Health Study. American Journal of Obstetrics & Gynecology. Apr;208(4):316.e1-. |
| Exclude | Wrong publication type (abstract) | Romieu I, Barraza-Villarreal A, Hern, ez-Cadena L, Escamilla-Nunez C, Sly P, et al. Supplementation with omega-3 fatty acids and atopy symptoms in infants: a randomized controlled trial. European respiratory society annual congress, berlin, germany, october 4-8. 2008 2008:[P2016]. |
| Exclude | Wrong publication type (abstract) | Scalabrin D, Mitmesser S, Birch E, Khoury J, Bean J, Harris C, et al. Lower incidence and less recurrence of allergic manifestations is observed in children who received docosahexaenoic acid/arachidonic acid in infancy via breast milk or supplemented formula. Allergy: european journal of allergy and clinical immunology Conference: 30th congress of the european academy of allergy and clinical immunology istanbul turkey Conference start: 20110611 conference end: 20110615 Conference publication: (varpagings). 2011 2011;66(94):711. |
| Exclude | Wrong intervention (FA levels) | Warstedt K, Furuhjelm C, Falth-Magnusson K, Fageras M, Duchen K. High levels of omega-3 fatty acids in milk from omega-3 fatty acid-supplemented mothers are related to less immunoglobulin E-associated disease in infancy. Acta Paediatrica. Nov;105(11):1337-47. |
| Exclude | Wrong outcome | Denburg JA, Hatfield HM, Cyr MM, Hayes L, Holt PG, Sehmi R, et al. Fish oil supplementation in pregnancy modifies neonatal progenitors at birth in infants at risk of atopy. Pediatric Research. Feb;57(2):276-81. |
| Exclude | Wrong outcome | D'Vaz N, Meldrum SJ, Dunstan JA, Lee-Pullen TF, Metcalfe J, Holt BJ, et al. Fish oil supplementation in early infancy modulates developing infant immune responses. Clinical & Experimental Allergy. Aug;42(8):1206-16. |
| Exclude | Wrong outcome | Jain J, Kern-Goldberger AR, Gyamfi-Bannerman C. Omega-3 supplementation in pregnancy and neonatal respiratory outcomes Mirella Mourad1. American journal of obstetrics and gynecology. 2018 2018;218(1):S96‐. |
| Exclude | Wrong outcome | Rago D, Rasmussen MA, Lee-Sarwar KA, Weiss ST, Lasky-Su J, Stokholm J, et al. Fish-oil supplementation in pregnancy, child metabolomics and asthma risk. EBioMedicine. Aug;46:399-410. |
|  |  |  |
